# Initial Experience of the Medtronic Aurora Extravascular Implantable Cardioverter in Children in a UK Centre

**DOI:** 10.1007/s00246-025-04031-z

**Published:** 2025-10-22

**Authors:** Georgia Spentzou, Sodiq Ayodeji Yaya, Palash Barman

**Affiliations:** 1 University of Bristol, Bristol, UK; 2 Pediatric Cardiology, Bristol Royal Hospital for Children, Bristol, UK; 3 Bristol Heart Institute, University Hospitals Bristol and Weston NHS Foundation Trust, Bristol, UK

**Keywords:** Cardiac defibrillator, Children, Pediatric, Aurora EV-ICD

## Abstract

Implantation of an implantable cardioverter-defibrillator (ICD) in childhood comes with many challenges due to implantation of a device early in life, the small patient size and ongoing growth. The Aurora EV-ICD is the smallest extravascular device with a novel implantation technique. Implantation of the Aurora EV-ICD was performed in three paediatric patients in our institution between September 2024 and February 2025. The ages of the patients were 6, 12 and 16 years. The weight range was 20 to 45 kg, and the height range was 121 to 161 cm. The procedure was performed under general anaesthesia. The implantation was uneventful in all cases. A 52 cm EV2401 lead was implanted using the provided tunnelling tool with excellent sensing and pacing parameters. The Aurora EV-ICD^TM^ MRI SureScan^TM^ system was placed in a subcutaneous pocket in the left axilla. Defibrillation testing was successful in 2 cases (after a single 30 J shock); ventricular fibrillation was not induced successfully in one of the cases. The routine 6-week post-operative ICD interrogation was satisfactory for all cases with stable sensed R wave measurements. The novel use of the Aurora EV-ICD may mark a significant development in the realm of ICDs for children by circumventing complications linked to transvenous leads, preserving vascular integrity and offering the smallest available extravascular ICD option. Our experience shows that this novel ICD can be implanted successfully in the pediatric population, but evidence on long-term functionality of the device in children is required.

## Introduction

Implantation of an implantable cardioverter-defibrillator (ICD) in childhood comes with multiple challenges due to implantation of a device early in life, the small patient size and ongoing growth. The subcutaneous ICD (S-ICD) is often the favoured approach to avoid the presence of a shock coil in the circulation when pacing is not required; however, its large size can be a limiting factor for the small patients and can lead to a poor cosmetic outcome and discomfort. The Aurora EV-ICD is a smaller device with a novel implantation technique. Its proximity to the heart enables delivery of anti-tachycardia pacing (ATP) [1]. It was FDA approved in 2023, but as it is a novel device, there were no published reports of its use in pediatrics at the time of implantation of the device in children in our institution.  Here we describe the initial experience of the Medtronic Aurora EV-ICD in the pediatric population in our institution.

## Methods

Following completion of training of a high-volume operator by Medtronic (PB), three pediatric cases were selected for implantation of the Aurora EV-ICD. The devices were implanted between September 2024 and February 2025. Cross-sectional imaging (cardiac MRI scan in 2 patients with a CT angiogram in one patient) was performed to assess their suitability. Informed consent was obtained for implantation of the Aurora EV-ICD. Consent was also obtained for implantation of an alternative ICD device in the event of unsuccessful implantation of the Aurora EV-ICD. All patients met criteria for ICD implantation as per the 2022 ESC Guidelines [2].

### Case 1

16-year-old female (weight 45 kg, height 161 cm) with a diagnosis of Catecholaminergic Polymorphic Ventricular Tachycardia and recorded bidirectional ventricular tachycardia despite optimal medical therapy (high dose nadolol and flecainide) and a left cardiac sympathetic denervation.

### Case 2

12-year-old female (weight 39 kg, height 161 cm) with a history of a ventricular fibrillation out-of-hospital arrest. No cause was identified on subsequent cardiac investigations or genetic testing (sudden cardiac death gene panel negative).

### Case 3

6-year-old female (weight 20 kg, height 121 cm) with a diagnosis of long QT syndrome due to a pathogenic calmodulin variant. The patient had presented with a ventricular fibrillation out-of-hospital arrest at 12 months and was subsequently implanted with a non-conventional ICD (transvenous sensing lead, subcutaneous shock coil placement in the thorax with the ICD generator in the abdominal wall). She had undergone multiple revisions for lead repositioning, and she presented with poor R wave sensing, requiring replacement of the ICD system.

### Implantation Technique

**Fig. 1:**
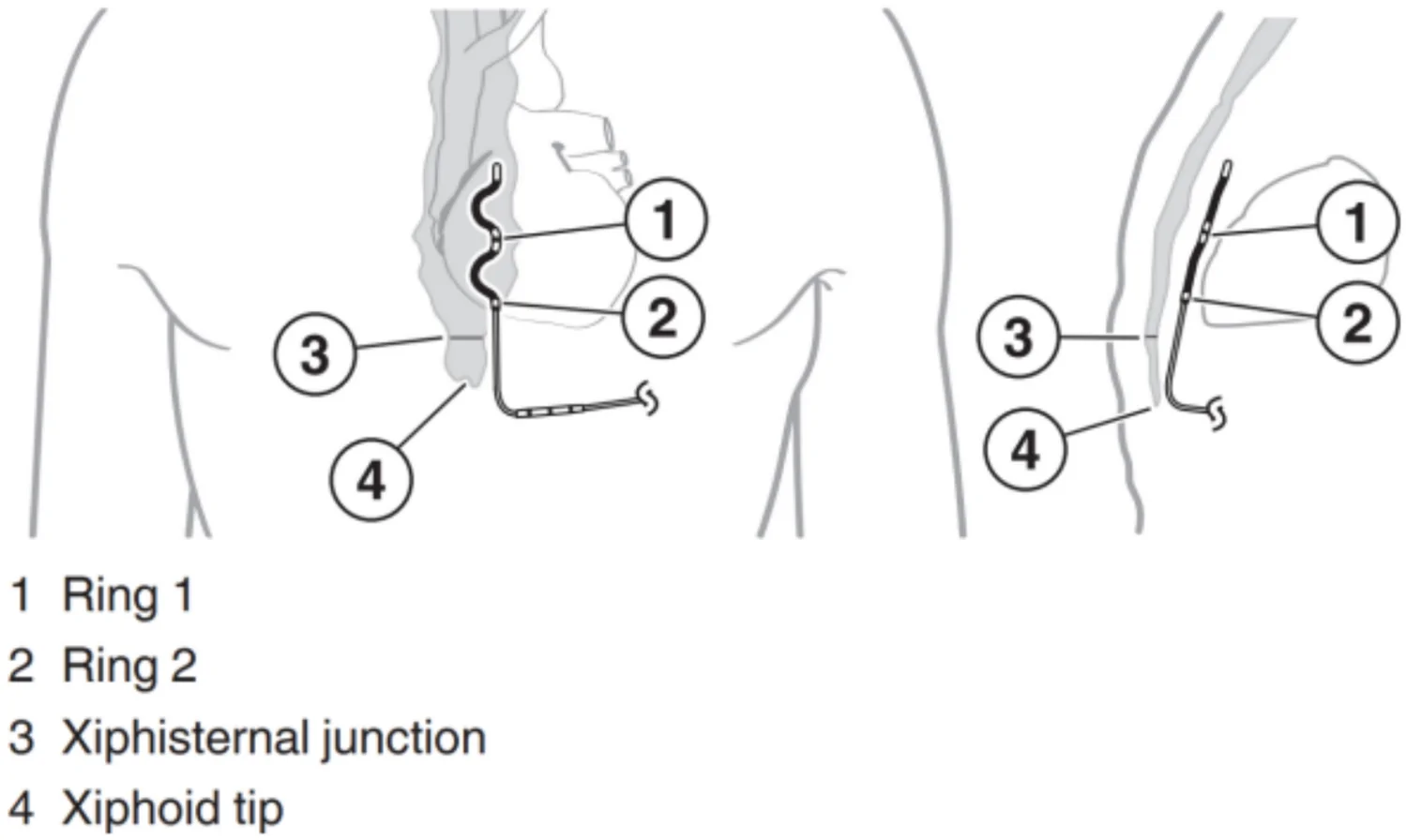
Recommended position of the lead behind the sternum (courtesy of Medtronic)

The Epsila EV™ MRI SureScan™ Model EV2401 lead has been designed with 2 electrodes (Ring 1 and 2) and 2 defibrillation coils. The distance between the tips of the lead to the Ring 2 electrode is 9 cm. Figure 1 shows the recommended position of the lead behind the sternum. Care should be taken to avoid implanting the Ring 2 electrode caudal to the xiphoid tip as that could lead to possible lead damage. In addition, if the lead covers a large portion of the right atrial mass, this may result in a large sensed p wave. In such a scenario, the position of the lead cannot be accepted as the discrimination algorithms of the device cannot overcome the issue of the large sensed atrial activity. We measured the length of the chest in the 2 younger patients and ensured it was at least 9 cm long as recommended by Medtronic. In addition, we performed cross-sectional imaging for all cases as part of pre-procedural planning to ensure an adequate length of sternum. The cross-sectional imaging was also useful in assessing the retrosternal space; if too wide, sensing issues may arise. Finally, the cross-sectional imaging allowed us to rule out a pectus deformity and the uncommon anatomical variant of both pleura meeting at the lower end of the sternum that could cause issues with the implantation, unless noted in advance in which case breath holding can allow safe tunneling.

General anaesthesia was provided to all patients. The recommended implantation technique by Medtronic was used. A Medtronic technical consultant supported all procedures. A horizontal costal margin incision was performed followed by dissection to the rectus fascia. The limited ossification of the xiphoid process made it harder to identify in the youngest patient with palpation. Separation of the diaphragmatic attachments was performed with blunt finger dissection which was somewhat harder than in adults, due to firmer tissues in children. In addition, the costal margins in children tend to be vertical creating a sharp angle, making separation of the diaphragmatic attachments slightly more challenging, further compounded by double gloving. Despite this, separation of the diaphragmatic attachments was performed successfully with blunt finger dissection as recommended by Medtronic in all cases.

The Epsila EV™ Model EAZ101 sternal tunnelling tool was advanced under fluoroscopic guidance using posteroanterior and lateral projections. A 52 cm lead was implanted in the recommended orientation (coils towards the right of the patient with the electrodes towards the left) and leftwards to the midline. The lead was connected to an Aurora EV-ICD™ MRI SureScan™ system which was placed in a subcutaneous pocket in the left axilla. Any excess lead was looped in the pocket under the device.

### Outcomes

**Fig. 2:**
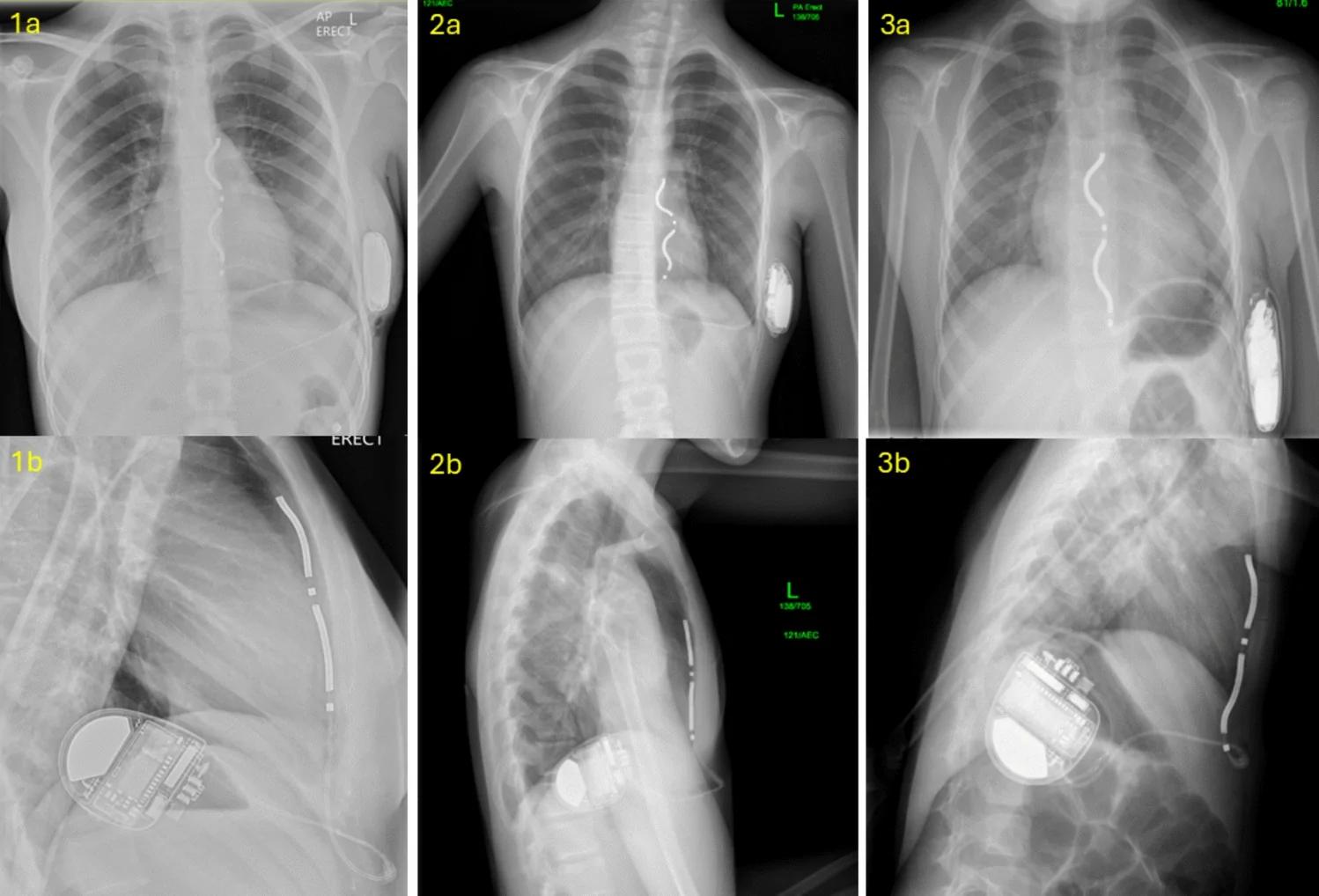
Posteroanterior chest x-rays for case 1 (**1a**), case 2 (**2a**) and case 3 (**3a**). Lateral projection for case 1 (**1b**), case 2 (**2b**) and case 3 (**3b**)

The implantation was uneventful in all cases. The second case required a second tunnelling attempt as the coil rotated after tunnelling to the pocket. The position of the lead and the ICD generator is shown in Fig. 2. The median fluoroscopy time was 6 min (range 3.4 to 7 min). The median procedural time was 121 min (range 97 to 243 min; case 3 required removal of the transvenous lead and previous ICD system which prolonged the procedure significantly). There was excellent sensing with no p wave oversensing. The final sensed R wave was between 3.5 and 4.6 mV for the 3 cases. Defibrillation testing was successful in 2 cases (after a single 30 J shock); for case 1, ventricular fibrillation was not induced successfully. ATP was programmed for all 3 cases.

The routine 6-week post-operative ICD interrogation was satisfactory for all cases. The sensed R wave was measured between 3.5 and 4.4 mV at follow-up. Case 1 has received appropriate and successful ICD therapy following the first post-operative ICD interrogation. There have been no wound complications and all patients have been satisfied with the cosmetic outcome.

## Discussion

The Medtronic Aurora EV-ICD is an MRI conditional, low profile device (33mls) which is the same size as the Medtronic transvenous single chamber ICDs. Its efficacy at delivering defibrillation therapy was demonstrated in the EV-ICD Pivotal and its follow-up study [1,3]. It can provide pause-prevention & post-shock pacing. Additionally, it can provide antitachycardia pacing (ATP) which is a unique feature compared to other extravascular ICDs. The effectiveness of the ATP of this device was demonstrated in the Pivotal study [3], where ATP successfully terminated 77% of episodes of ventricular tachycardia. Its small size, the ability to provide ATP and the secure position in the retrosternal space, makes this device an attractive option for the pediatric patients.

The selection of ICD systems for pediatric patients is often influenced by characteristics such as the small size of their hearts and vessels, anatomical variations, and a propensity for heightened physical activity. The selection of the device must be meticulously considered in a population that outlives its ICD device. There is an obvious need to address the frequent problems associated with transvenous (TV) ICD in the children, that are associated with a high incidence of venous occlusion or thrombosis, lead failure and the long-term risk of infection, cardiac perforation, and interference with tricuspid valve function [4,5]. Surgical implantation techniques on the epicardial surface can successfully mitigate against certain complications linked to TV-ICD. However, they are associated with significant morbidity and a high rate of reoperation for lead or device-related complications [5,6]. The S-ICD has been widely used in the pediatric population with good outcomes [7,8]. Nevertheless, its use is mostly limited to the adolescent population or the older primary school age children, as the large size of the device renders it unfavourable for the very small children.

The Aurora EV-ICD remains a relatively novel device with limited long-term data. There has been one main cohort of patients enrolled in the Pivotal study with follow-up studies on long-term results in the same patient cohort [9], quality of life [10], detection of ventricular tachyarrhythmia events during follow-up [11] and lead removal experience [12]. A recent systematic review identified only small additional studies published in the last 4 years with patient numbers less than 40 each [13]. In terms of implanting this device in children, there has been one case report published of the use of this device in a 2-year-old patient [14]. The procedure was performed without any complications and the patient has since received successful defibrillation for ventricular tachycardia by the device. There has also been a case report in the use of this device in an 18-year-old athlete [15]. The exact anthropometrics were not provided, but the authors reported that the patient was of a slender build. More recently, a case series of 7 children, aged 11 to 16 years, was published demonstrating successful implantation in all cases with no complications [16]. Interestingly, the authors elected to place a subcutaneous abdominal loop to accommodate body growth. This was not performed in our patients, but the need for this will be assessed during longterm follow-up.

When implanting an EV-ICD in children, one should be alert to the anatomical differences between adults and children. As in our cases, the firmer tissues, vertical costal margins and incomplete ossification of the xiphoid process, may make the separation of the diaphragmatic attachments more challenging. However, as the recently published case series and ours demonstrate, this element of the procedure can be achieved safely with blunt finger dissection as recommended. The technique may need to be modified to allow for future growth by inserting a subcutaneous loop. According to the authors’ local experience from the S-ICDs in children, this is not always necessary, but longterm follow-up of the children implanted with an EV-ICD will clarify whether a subcutaneous loop should be considered.

As published data in particularly young children are very limited (only one case report of a 2-year-old and one 6-year-old child included in our case series), the suitability of this device in very young children remains uncertain. Careful pre-procedural planning with cross-sectional imaging is important. In addition, an honest discussion with the parents or carers of such young children with regard to lack of information on long-term performance and outcomes of this device should take place before any young child is put forward for this. Despite the uncertainties, the EV-ICD may still present as a favoured option for some parents compared to the alternatives.

Finally, cross-sectional imaging plays an important role in patient selection and procedural planning in children to increase the chances of successful implantation without p wave oversensing, with adequate R wave sensing and to rule out anatomical variants that may complicate the procedure.

## Conclusions

The novel use of the Aurora EV-ICD may mark a significant development in the realm of ICDs for children by circumventing complications linked to transvenous leads, preserving vascular integrity and offering the smallest available extravascular ICD option. Our experience shows that this novel ICD can be implanted successfully in the pediatric population. There is clearly a need for long-term outcome data of this device^9^. One crucial topic for further study, specific to the pediatric population, is whether the children's growth will impact the device's functionality. However, the Aurora EV-ICD presents so far, an attractive option for children and adolescents.

## Competing interests

The authors declare no competing interests.

## Data Availability

No datasets were generated or analysed during the current study.
